# Differences in the intestinal microbiota and association of host metabolism with hair coat status in cattle

**DOI:** 10.3389/fmicb.2024.1296602

**Published:** 2024-04-22

**Authors:** Donglin Wu, Pengfei Zhao, Chunjie Wang, Simujide Huasai, Hao Chen, Aorigele Chen

**Affiliations:** ^1^College of Animal Science, Inner Mongolia Agricultural University, Hohhot, China; ^2^College of Veterinary Medicine, Inner Mongolia Agricultural University, Hohhot, China

**Keywords:** cattle, hair coat, intestinal microbiota, interplay, metabolomics

## Abstract

**Introduction:**

The hair coat status of cattle serves as an easily observed indicator of economic value in livestock production; however, the underlying mechanism remains largely unknown. Therefore, the objective of the current study was to determine differences in the intestinal microbiota and metabolome of cattle based on a division of with either slick and shining (SHC) or rough and dull (MHC) hair coat in Simmental cows.

**Methods:**

Eight SHC and eight MHC late-pregnancy Simmental cows (with similar parities, body weights, and body conditions) were selected based on their hair coat status, and blood samples (plasma) from coccygeal venipuncture and fecal samples from the rectum were collected. The intestinal microbiota (in the fecal samples) was characterized by employing 16S rRNA gene sequencing targeting the V3–V4 hypervariable region on the Illumina MiSeq PE300 platform, and plasma samples were subjected to LC–MS/MS-based metabolomics with Progenesis QI 2.3. Plasma macromolecular metabolites were examined for differences in the metabolism of lipids, proteins, mineral elements, and hormones.

**Results:**

Notable differences between the SHC and MHC groups related to host hair coat status were observed in the host metabolome and intestinal microbiota (*P* < 0.05). The host metabolome was enriched in histidine metabolism, cysteine and methionine metabolism, and purine metabolism in the SHC group, and the intestinal microbiota were also enriched in histidine metabolism (*P* < 0.05). In the MHC group, the symbiotic relationship transitioned from cooperation to competition in the MHC group, and an uncoupling effect was present in the microbe–metabolite association of intestine microbiota–host interactions. The hubs mediating the relationships between intestinal microbiota and plasma metabolites were the intestinal bacterial genus *g__norank_f__Eubacterium_coprostanoligenes_group*, plasma inosine, triiodothyronine, and phosphorus, which could be used to differentiate cows’ hair coat status (*P* < 0.05).

**Conclusion:**

Overall, the present study identified the relationships between the features of the intestinal microbiota and host hair coat status, thereby providing evidence and a new direction (intestine microbiota–host interplay) for future studies aimed at understanding the hair coat status of cattle.

## 1 Introduction

The condition of cattle’s hair coat serves as a readily observable economic indicator in livestock farming, and it has been employed in animal production practices since the 1960s (Turner and Schleger, 1960; Pan, 1964). Hair coat status reveals cattle’s health, metabolism, and productive performance. For instance, slick-haired Holstein cows exhibit better thermoregulation and lesser milk production declines in summer than non-slick ones (Dikmen et al., 2014). Additionally, the presence of the slick hair mutation helps counter fescue toxicosis effects and improves beef cattle heifers’ reproduction (Poole et al., 2019). The significance of hair coat status and its impact on cattle are well-known; however, the underlying mechanisms that influence hair coat status remain largely unexplored. Recent research has revealed that gut microbiota, operating in symbiosis with their hosts, can impact hair status in humans (Mahmud et al., 2022) and in mice (Hayashi et al., 2017; Nam et al., 2021). This information may provide insight for further exploration into the mechanisms influencing hair coat status in cattle.

Hair coat status is closely connected to the physiological metabolism of cattle. As a derivative of the skin, hair growth is regulated by nutrient metabolism (Lv et al., 2020). A prime example of nutrient metabolism’s impact on hair coat is how diet supplementation enhances cashmere fiber characteristics in goats (Ma et al., 2012). Hormones, such as growth hormones (Vissenberg et al., 2020) and sex hormones (Grymowicz et al., 2020), may also play roles in hair growth. These hormones exert their influence by binding to their corresponding receptors in the dermal papilla cells of the hair follicle (Rosenberg et al., 2021). Environmental factors that cause psychological stress can also affect hair status. For instance, hair graying and reversal have been linked to life stress in humans (Cavan et al., 1993). Additionally, other components that are directly involved in hair formation can influence hair coat status. For example, zinc levels in hair are associated with body composition and growth in children (Cavan et al., 1993), while copper and zinc deficiencies can lead to diffuse alopecia and kinky hair syndrome (DiBaise and Tarleton, 2019). In cattle, copper deficiency can result in changes in hair color, texture, growth, and appearance. Severe copper deficiency may even cause hair coat loss (Underwood, 1977; Engle et al., 2016).

In recent years, there has been a surge in studies utilizing metabolomics, highlighting the value of this high-throughput technique for understanding the metabolic mechanisms underlying host traits (Goldansaz et al., 2017). Moreover, metabolomics can reveal metabolic pathways, provide high-throughput characterization of metabolites, and identify small-molecule metabolites involved in various biological processes (Hao et al., 2021). Utilizing multi-omics approaches, such as 16S rRNA gene sequencing, metagenomics, and meta-transcriptomics, researchers have obtained a wealth of data and gained new insights into host and intestinal microbiota metabolism, as well as the intricate interplay between microbiota and host that affects diverse economic traits (Wishart, 2019; Huang et al., 2021) and feeding (Lin et al., 2021; Huang et al., 2022). However, these multi-omics techniques have not yet been applied to better understand the relationship between hair coat status and intestinal microbiota in cattle.

The interaction between the intestinal microbiota and host has been reported to influence hair status in humans, highlighting the existence of an intestinal microbiota–skin axis that impacts various skin conditions, including alopecia areata (Mahmud et al., 2022). Confirming this, earlier investigations have found that dysbiosis of intestinal microbiota induced alopecia through the overgrowth of *Lactobacillus murinus* in mice (Hayashi et al., 2017), and that *Lactobacillus paracasei* HY7015 fostered hair growth within a telogenic mouse model (Nam et al., 2021). Additionally, the intestinal microbiota plays a role in nutrient digestion and utilization, factors that eventually affect hair health (Yadav and Jha, 2019). A prominent example is the digestion of fiber in both the rumen of ruminants and the lower guts of non-ruminants, which yields short-chain fatty acids that supply energy to the host animals and contribute to the maintenance of their overall health (Guilloteau et al., 2010). Importantly, intestinal microbiota serves as a major regulator of circulating hormones, as it works to deconjugate and activate these hormones (Menon et al., 2013). Conversely, hormones direct the modulation of microbiota metabolism through their hormone receptors (Yoon and Kim, 2021). Recently, evidence of the relationship between host hormones and intestinal microbiota has been observed in Simmental cows (Wu et al., 2022).

The influence of intestinal microbiota on cattle hair coat status remians largely unexplored. Consequently, the objective of this study was to identify the relationship between cattle hair coat status and intestinal microbiota by examining hair coat status classified as either slick and shining (SHC) or rough and dull (MHC) in a late-pregnancy Simmental cow model. We hypothesized that the intestinal microbiota would be connected to host metabolism and thereby affect the hair coat status. In order to evaluate this hypothesis, we conducted analyses of: (1) plasma metabolites of nutrients, hormones, and mineral elements; (2) plasma metabolome through non-targeted metabolomics analysis; and (3) intestinal microbiota using 16S rRNA gene sequencing.

## 2 Materials and methods

### 2.1 Animals and experimental design

All procedures related to the care and management of animals received approval from the Institutional Animal Care and Use Committee at Inner Mongolia Agricultural University Approval Number: NND2023100 (Hohhot, China). Sixteen healthy Simmental cows were selected based on hair coat status as slick and shining (SHC, *n* = 8) or rough and dull (MHC, *n* = 8) during the late pregnancy period. These cows were: multiparous non-lactating parities (three parities), had an average body weight of 557.81 ± 27.91 kg, and a body condition score of 6 (on a scale of 1 to 9, where 1 = very thin and 9 = very fat). The cows were housed in a free-stall barn and fed with total mixed rations at 9:00 and 14:00 daily, and water was freely available.

### 2.2 Sample collection

Blood samples were obtained through coccygeal venipuncture at 08:00 and collected into tubes preloaded with heparin sodium (Hebei Kang Wei Shi Medical Equipment Co., Ltd., Shijiazhuang, China). These tubes were swiftly cooled on ice and then subjected to centrifugation at 1800 × *g* for 10 min. Following this process, the plasma was isolated, transferred to the laboratory, and stored at −80°C for subsequent analysis. Fecal samples were collected from the rectum by hand using sterile gloves, immediately transferred into sterile and pyrogen-free centrifuge tubes, frozen in liquid nitrogen, and stored at −80°C for further analysis. Representative samples of the total mixed rations (1000 g) were obtained and pooled. The total mixed ration samples were analyzed for dry matter, crude protein, ether extract, neutral detergent fiber, acid detergent fiber, phosphorus, and calcium (Wu et al., 2022). The chemical composition of the total mixed rations is displayed in Supplementary Table 1.

### 2.3 Plasma parameters analysis

The plasma was analyzed for glucose, triglycerides (TG), total cholesterol, low-density lipoprotein cholesterol, high-density lipoprotein cholesterol (HDL-C), total protein, albumin, aspartate aminotransferase (AST), alanine aminotransferase (ALT), total bilirubin, creatinine, urea nitrogen, creatine kinase, and lactate dehydrogenase using corresponding commercial kits (AngleGene Biotechnology Co. Ltd., Nanjing, China) following the manufacturer’s instructions. Analyses were conducted automatically by spectrophotometry (Shimadzu 2100, Kyoto, Japan) (Weber et al., 2013). The major and trace mineral elements evaluated were phosphorus, calcium, magnesium, zinc, iron, and copper. Briefly, the plasma samples were digested with nitric acid and perchloric acid, and then the digests were diluted to an appropriate concentration with ultra-pure water (Gong et al., 2014). A standard regression equation was established with appropriate concentrations for each mineral element according to the reference materials (NCS Testing Technology Co. Ltd., Beijing, China). The element samples were analyzed on an inductively coupled plasma emission spectrometer (iCAP6300, Thermo Fisher Scientific Inc., Waltham, MA, USA). Triiodothyronine (T3), tetraiodothyronine, melatonin, progesterone, estradiol 2, prolactin, growth hormone, insulin-like growth factor 1, insulin, and cortisol were measured using corresponding commercial kits (AngleGene Biotechnology Co. Ltd, Nanjing, China) in accordance with the manufacturer’s instructions using an enzyme-linked immunosorbent assay (ELISA) on a microplate reader (Biotek Synergy H1, BioTek Instruments, Inc., Winooski, VT, USA) as previously reported (Wu et al., 2022).

### 2.4 Metabolomics analysis of plasma samples and data processing

A non-targeted metabolomics analysis was performed on the plasma samples utilizing LC-MS/MS. Free metabolites were isolated conforming to the extraction techniques highlighted in previous research (Chen H. et al., 2021). To ensure consistent quality and performance of the analysis, equal volumes of plasma samples were combined to create a pooled quality control (QC) sample. This QC sample was intermittently injected into the system to monitor the stability of the analysis. Chromatographic separation of the metabolites was carried out on a Thermo UHPLC system, with the detailed procedure described in earlier studies (Chen H. et al., 2021). Following UPLC-TOF/MS analyses, the initial data were imported into Progenesis QI 2.3 (Non-linear Dynamics, Waters) for preprocessing tasks including baseline filtering, peak detection, and alignment. Subsequently, metabolites were identified through searches in the Human Metabolome Database (HMDB), the Metlin Database, and the Majorbio Database (Liu et al., 2022). All data comparisons between the SHC and MHC groups were conducted using orthogonal partial least squares discriminant analysis (OPLS-DA) in conjunction with Student’s *t*-test, employing the following screening criteria: variable importance in the projection (VIP) values >1.0 and *P* < 0.05 to identify metabolites with significant differential abundance between the two groups. The significantly differentially abundant metabolites were further analyzed by summarizing and mapping them to their respective biochemical pathways through metabolic enrichment and pathway analysis, utilizing a database search (KEGG).^1^ The impact of the metabolic pathways and metabolite set enrichment was assessed using the “stats” package in R and the “scipy” package in Python, respectively. Significantly enriched KEGG metabolic pathways were determined based on a *P*-value threshold of <0.05.

### 2.5 16S rRNA gene sequencing and microbial data processing

Genomic DNA from the fecal samples was extracted using the hexadecyl trimethyl ammonium bromide (CTAB) method. Subsequently, the DNA extracts were subjected to quality checks on 1% agarose gels, and the DNA concentration and purity were assessed using a NanoDrop 2000 UV-vis spectrophotometer (Thermo Scientific, Waltham, MA, USA). For microbial community analysis, the V3-V4 hypervariable regions of the microbial 16S rRNA genes were amplified and sequenced using the Illumina MiSeq PE300 platform (Shanghai Majorbio Bio-Pham Technology Co., Ltd, Shanghai, China) with the primer pairs 338F (5′-ACTCCTACGGGAGGCAGCA-3′) and 806R (5′-GGACTACHVGGGTWTCTAAT-3′). PCR conditions were consistent with those detailed in a previous study (Wu et al., 2022). The raw 16S rRNA gene sequences were demultiplexed, underwent quality filtration using fastp (version 0.20.0), and merged using FLASH (version 1.2.7). Qualified sequences were filtered according to previously reported criteria (Wu et al., 2022). Operational taxonomic units (OTUs) were clustered at a 97% similarity cutoff level using Uparse. Chimeric sequences were identified and subsequently removed utilizing Usearch (version 7.0) as described previously (Edgar, 2013). Taxonomic assignments for each OTU were made by applying the RDP Classifier algorithm to classify their representative sequences against the Silva 16S rRNA database (Silva v138), using a confidence threshold of 70%, following the methodology outlined previously (Wang et al., 2007). Venn diagrams were constructed to illustrate the core microbiota at the OTU level. Alpha diversity was evaluated by the richness (Sob index), community diversity (Shannon index), and evenness (Shannon evenness index). Beta diversity was analyzed using the unweighted and weighted UniFrac distances followed by the analysis of similarities (ANOSIM) and was visualized using non-metric multidimensional scaling (NMDS). We applied Linear Discriminant Analysis Effect Size (LEfSe) to pinpoint taxa that underlie distinctions between the two metadata classes, spanning phylogenetic levels from genus to phylum. A linear discriminant analysis score filter value of 3 was employed as the default setting. In addition, the differences in bacteria between the two groups at the genus level were tested by Student’s *t*-test. Predicted functional profiles of microbial communities were generated through the application of the phylogenetic investigation of communities by reconstruction of unobserved states (PICRUSt) technique (Langille et al., 2013). These predicted functional composition profiles were subsequently aggregated into level 3 KEGG database pathways, with differences in bacterial functions assessed using the Student’s *t*-test.

### 2.6 Statistical analysis

Before we started the analysis, we conducted a screening process on all data to confirm normality and variance homogeneity using SPSS Statistics (version 24.0; IBM Corp., Armonk, NY, USA). If any data didn’t conform to the assumptions of normality, we applied logarithmic transformations [log10(x)] to create a more uniform distribution. For this study, we treated individual cows as the experimental units (*n* = 8). Data related to plasma indices were analyzed using the Student’s *t*-test in SPSS Statistics. The co-occurrence probabilities between microbes and microbes or between microbes and metabolites were tested (Spearman correlation; *r* > 0.5, *P* < 0.05) using Networkx (Shanghai Majorbio Bio-Pharm Technology Co. Ltd.). Lastly, we determined whether the assessment of the cows’ biomarkers of intestinal microbiota could improve our ability to predict the cows’ hair coat status using the following approaches (Alderete et al., 2021): (1) the data were divided into two categories for hair coat status; and (2) the area under the receiver operating characteristic (AUROC) curves was tested with the SHC as a control group and with the MHC as the treated group in GraphPad Prism (version 8.0.2; GraphPad Software, Inc., San Diego, CA, USA). GraphPad Prism was also used to construct the figures. Statistical significance was set at *P* < 0.05.

## 3 Results

### 3.1 Macromolecular metabolites in the plasma

Table 1 displays the values for macromolecular metabolites in the plasma of cows with different hair coat statuses. In comparison to the MHC group, cows in the SHC group demonstrated a relatively higher metabolism for lipids (TG and HDL-C), protein, liver function (AST and AST/ALT), cardiac function and energy metabolism (creatine kinase and lactate dehydrogenase), mineral element (phosphorus), nutritional elements (T3, tetraiodothyronine, and melatonin), and reproductive (progesterone) hormone metabolism (*P* < 0.05 or *P* < 0.01). No significant differences were observed in glucose metabolism, renal function, or stress hormones between the two groups (*P* > 0.05).

**TABLE 1:** Differential plasma macromolecule metabolites in cows exhibiting slick and shining (SHC) or rough and dull (MHC) hair coat status.

Item	SHC group	MHC group	SEM	*P*-value
**Glucose and lipid metabolism**				
Glucose, mmol/L	2.933	2.613	0.219	0.166
Triglycerides, mmol/L	0.223	0.163	0.028	0.052
Total cholesterol, mmol/L	2.255	1.783	0.239	0.068
Low-density lipoprotein cholesterol, mmol/L	0.375	0.279	0.058	0.118
High-density lipoprotein cholesterol, mmol/L	1.603	1.279	0.146	0.043
**Protein metabolism and liver function**				
Total protein, g/L	54.66	45.73	3.24	0.016
Albumin, g/L	28.94	26.87	2.17	0.356
Aspartate aminotransferase (AST), U/L	57.91	52.67	4.25	0.238
Alanine aminotransferase (ALT), U/L	26.32	17.67	2.63	0.005
AST/ALT	2.223	3.211	0.397	0.026
Total bilirubin, umol/L	0.474	0.506	0.063	0.621
**Renal function**				
Creatinine, umol/L	84.67	78.39	5.87	0.303
Urea nitrogen, mmol/L	4.910	4.339	0.391	0.166
**Energy metabolism and cardiac function**				
Creatine kinase, U/L	166.98	116.27	14.30	0.003
Lactate dehydrogenase, U/L	1121.72	973.89	68.29	0.048
**Mineral element metabolism**				
Phosphorus, μg/mL	220.25	178.63	15.55	0.018
Zinc, μg/mL	3.161	2.685	0.279	0.111
Iron, μg/mL	17.10	17.51	0.84	0.638
Calcium, μg/mL	136.40	119.09	11.63	0.159
Magnesium, μg/mL	85.80	78.57	6.66	0.296
Copper, μg/mL	1.833	1.647	0.118	0.135
**Nutritional, reproductive, and stress hormone metabolism**				
Triiodothyronine ng/mL	1.519	1.443	0.036	0.056
Tetraiodothyronine, ng/mL	64.01	57.73	3.01	0.056
Melatonin, pg/mL	127.78	114.29	7.01	0.075
Estradiol 2, pmol/L	22.65	20.35	1.75	0.209
Progesterone, ng/mL	30.35	27.06	1.43	0.037
Prolactin, uIU/mL	2278.39	2438.94	122.96	0.213
Growth hormone, ng/mL	38.51	34.05	3.40	0.211
Insulin-like growth factor 1, ng/mL	17.56	15.26	1.98	0.265
Cortisol, ng/mL	59.61	70.50	5.45	0.098
Insulin, uIU/mL	49.22	41.67	4.26	0.187

SEM, standard error of the means, *n* = 8; AST, aspartate aminotransferase; ALT, alanine aminotransferase. Differences were defined as significance with *P* < 0.05.

### 3.2 Differential metabolites screening and analysis of host plasma

**FIGURE 1:**
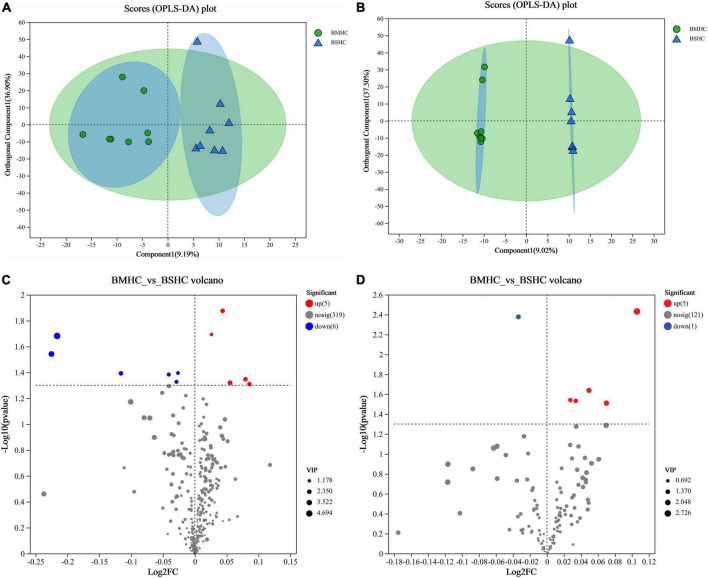
Identification of differential small-molecule metabolites and enriched metabolic pathways in the blood (plasma) metabolome of cows with slick and shining (BSHC) or rough and dull (BMHC) hair coat status. *n* = 8. Score plots of the orthogonal partial least squares discriminant analysis (OPLS-DA) in positive **(A)** and negative **(B)** ion mode. Visualization of significantly differential metabolites were visualized through volcano plots in positive **(C)** and negative **(D)** ion mode.

**FIGURE 2:**
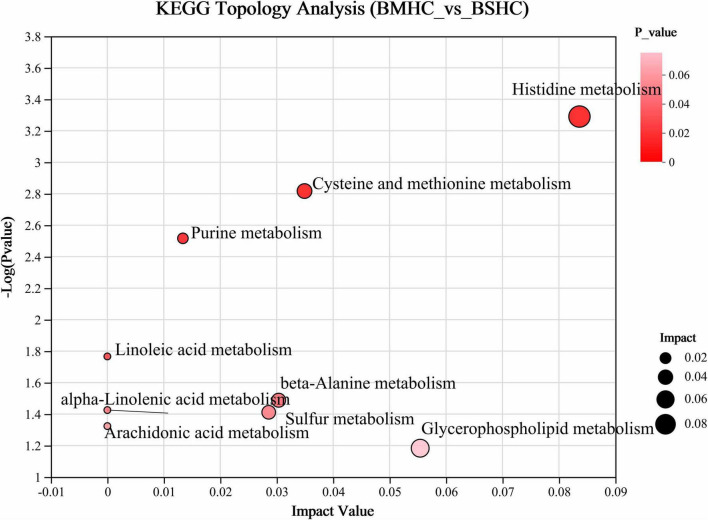
Enrichment analysis of differential metabolic pathways within the host metabolome (plasma) of cows with slick and shining (BSHC) or rough and dull (BMHC) hair coat status. Significant and impact pathways were identified using a significance value (*P* < 0.05) and KEGG topology analysis (impact value > 0).

We conducted a metabolomic analysis of blood plasma small-molecule metabolites for the BSHC and BMHC groups. To validate the distinct metabolite profiles between these two groups, we constructed OPLS-DA score plots in both positive and negative ion modes. Figures 1A, B visually depict the multivariate analysis. Importantly, the majority of plasma samples from the cows fell within the 95% confidence ellipses in the score plots. The validity of the OPLS-DA model was assessed using R2Y and Q2 values. In positive ion mode, the R2Y values for plasma samples were 0.865, while in negative ion mode, they reached 0.991. To assess the robustness and predictive capability of our models, we employed a sevenfold cross-validation method with permutations, and Q2 intercept values were computed after 200 permutations. The results are presented in Supplementary Figure 1. The permutation test results for the Q2 intercepts yielded values of −0.013 for positive plasma samples and −0.173 for negative plasma samples. Both positive and negative datasets exhibited distinct separation and discrimination between the BSHC and BMHC groups, underscoring the utility of the OPLS-DA model in identifying differences between the two groups. We analyzed a total of 457 metabolites in the plasma of cows, with 330 in positive ion mode and 127 in negative ion mode. Significantly differential metabolites between the two groups were identified from the pool of all identified metabolites using the Student’s *t*-test (*P* < 0.05) and a criterion of OPLS-DA model VIP > 1.0. We visualized the significantly differential metabolites using volcano plots (as shown in Figures 1C, D for both positive and negative ion modes). The figures distinctly illustrate the substantial differences in numerous plasma metabolites between the two groups, with 17 metabolites exhibiting significant differences (11 in positive ion mode and 6 in negative ion mode). A detailed list of these significantly differential metabolites between the two groups can be found in Table 2. The metabolites included organic acids and derivatives, lipids and lipid-like molecules, organoheterocyclic compounds, nucleosides, and nucleotides and analogs. The staple metabolic pathways were histidine metabolism, purine metabolism, cysteine and methionine metabolism, bile secretion, ABC transporters, glycerophospholipid metabolism, linoleic acid metabolism, beta-alanine metabolism, alpha-linolenic acid metabolism, arachidonic acid metabolism, choline metabolism in cancer, retrograde endocannabinoid signaling, and biosynthesis of amino acids (Table 2). As for these metabolic pathways (KEGG topology analysis), the significantly different (*P* > 0.05) and significant impact (impact value > 0) pathways were histidine metabolism, cysteine and methionine metabolism, and purine metabolism, and each was enriched in the SHC group (Figure 2). The metabolites that were enriched in these significantly different metabolic pathways were uric acid, o-acetylserine, urocanic acid, 1-aminocyclopropane-1-carboxylic acid, inosine, and carnosine (Table 2), and those metabolites were selected as biomarkers of the small-molecule metabolites of the host metabolome.

**TABLE 2:** Differential metabolites and their corresponding enriched metabolic pathways identified in the plasma metabolome of the host.

Metabolite	ID	VIP	FC	*P*-value	RT	M/Z	KEGG pathway description
LysoPC [20:3 (8Z, 11Z, 14Z)]	pos_89	2.497	0.962	0.048	7.867	546.4	–
Prolylhydroxyproline	pos_124	1.216	0.982	0.020	0.862	229.1	–
PC [18:0/20:4 (8Z, 11Z, 14Z, 17Z)]	pos_801	2.213	0.970	0.013	9.372	810.6	MP^1^
Thr Gly	pos_1680	1.724	1.029	0.041	0.783	177.1	–
Uric acid	pos_1720	2.009	0.943	0.049	0.921	169.0	Bile secretion; Purine metabolism
O-Acetylserine	pos_1745	1.632	1.020	0.047	1.082	189.1	MP^2^
Urocanic acid	pos_1758	2.135	0.946	0.045	1.210	139.1	Histidine metabolism
Cysteinyl-Threonine	pos_1821	3.736	1.170	0.029	1.488	205.1	–
Tsugaric acid C	pos_4858	4.694	1.161	0.021	5.688	497.4	–
Tyrosyl-Proline	pos_5687	2.189	1.083	0.040	2.116	317.1	–
1-Aminocyclopropane-1-carboxylic acid	pos_6152	1.465	1.018	0.040	0.921	102.1	Cysteine and methionine metabolism
Corchorifatty acid F	neg_332	1.198	0.981	0.029	6.549	327.2	–
6-(hydroxymethyl)-7-methoxy-2H-chromen-2-one	neg_1019	2.726	0.929	0.004	1.065	187.0	–
Isoleucyl-Hydroxyproline	neg_1161	1.721	0.952	0.031	1.636	243.1	–
Tavulin	neg_5693	1.298	0.977	0.029	4.585	285.1	–
Inosine	neg_6986	1.430	1.024	0.004	1.523	303.1	ABC transporters; Purine metabolism
Carnosine	neg_7343	1.599	0.966	0.023	0.630	225.1	Histidine metabolism; beta-Alanine metabolism

Differences in compared data were defined as significant with *P* < 0.05; *n* = 8. FC, fold change (BSHC/BMHC); RT, retention time; VIP, variable importance in projection.

^1^Metabolic pathways: glycerophospholipid metabolism; arachidonic acid metabolism; linoleic acid metabolism; alpha-linolenic acid metabolism; choline metabolism in cancer; retrograde endocannabinoid signaling.

^2^Metabolic pathways: sulfur metabolism; cysteine and methionine metabolism; carbon metabolism; biosynthesis of amino acids; sulfur relay system.

### 3.3 Diversity analysis of intestinal microbiota

**FIGURE 3:**
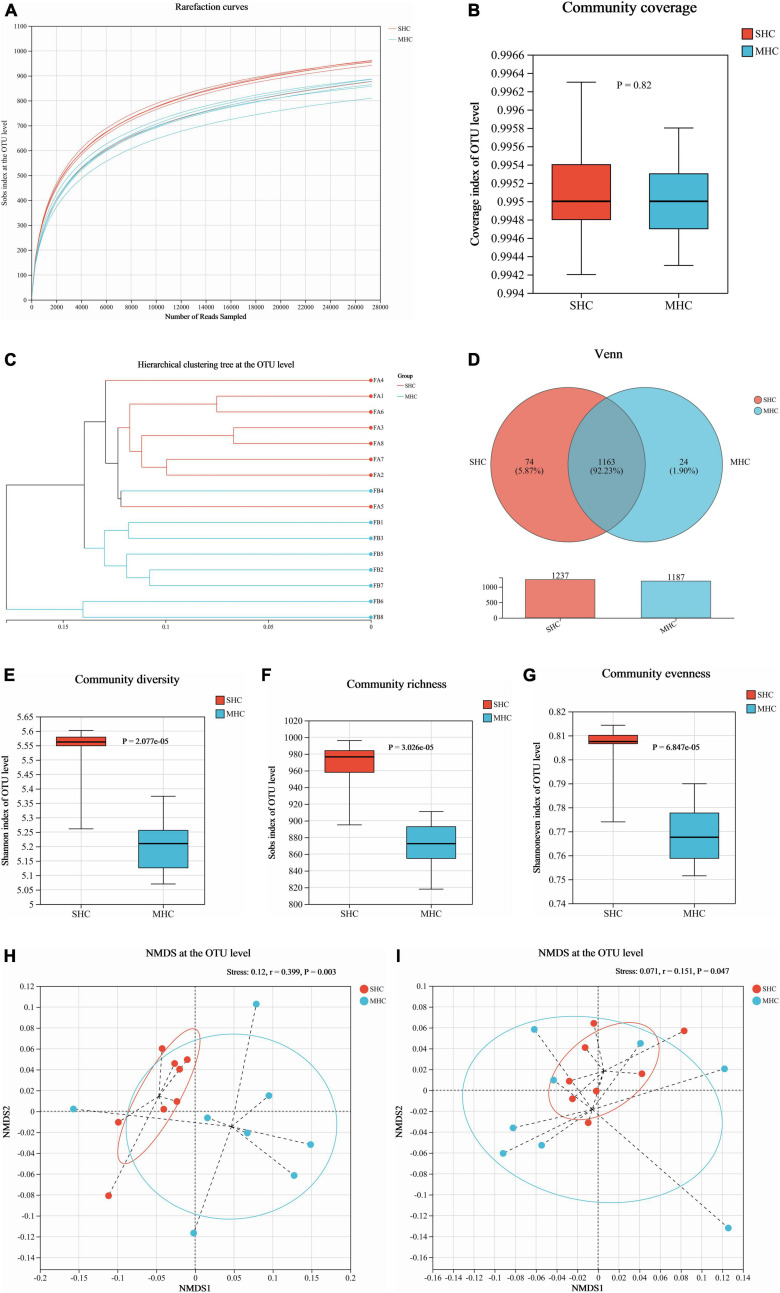
Assessment of intestinal microbiota diversity in cows with slick and shining (SHC) or rough and dull (MHC) hair coat status. Rarefaction curves **(A)**, sequencing depth **(B)**, hierarchical clustering tree **(C)**, and Venn diagram **(D)** at the OTU level based on sequencing data. Alpha diversity measured through community diversity (Shannon index) **(E)**, richness (Sob index) **(F)**, and evenness (Shannon even index) **(G)**. Beta diversity evaluated using the weighted **(H)** and unweighted **(I)** UniFrac distance followed by ANOSIM and visualized using non-metric multidimensional scaling (NMDS). Differences were defined as significance with *P* < 0.05; *n* = 8.

We acquired a total of 781,866 clean reads from the rectal fecal samples (Supplementary Table 2). The rarefaction curves (depicted in Figure 3A) and Goods coverage (≥ 99.5%, as illustrated in Figure 3B) offered assurance that each sample’s microbial diversity was adequately encapsulated with our chosen sequencing depth. Furthermore, there was no discernible disparity in sequencing depth between the two groups. The SHC group’s readings exposed a higher count of detected OTUs relative to those of the MHC group (Figure 3D). For fields such community diversity (Shannon index; Figure 3E, *P* < 0.001), community richness (Sob index; Figure 3F, *P* < 0.001), and community evenness (Shannon evenness index; Figure 3G, *P* < 0.001), the SHC group experienced a manifestation of higher alpha diversity indices at the OTU level of intestinal microbiota compared to the MHC group. Moving forward, the hierarchical clustering tree pinpointed instances of clustering in the majority of the samples from each group, indicating a decreased level of microbial similarity between groups (Figure 3C). A substantial dissimilarity was evident in the intestinal microbiota composition between the two groups, as indicated by the beta diversity indices employing the unweighted UniFrac distance (Stress = 0.120, *r* = 0.399, *P* < 0.01; Figure 3H) and the weighted UniFrac distance metrics (Stress = 0.071, *r* = 0.151, *P* < 0.05; Figure 3I).

### 3.4 Differential structure and function analysis of intestinal microbiota

**FIGURE 4:**
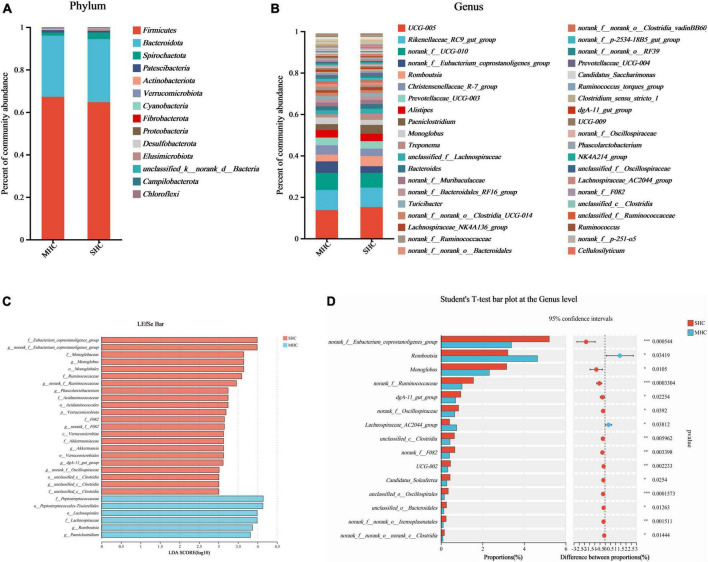
Comparative taxonomic analysis of intestinal microbiota in cows with slick and shining (SHC) or rough and dull (MHC) hair coat status. Intestinal microbiota composition at the phylum **(A)** and genus **(B)** levels. Differential species of intestinal microbiota from phylum level to genus level using LEfSe bar **(C)** and the Student’s *t*-test **(D)**. No distinguishing phyla were found, but fifteen disparate bacterial genera were detected via the Student’s *t*-test. Differences were defined as significance with *P* < 0.05; *P < 0.05; **P < 0.01; ***P < 0.001.

The abundance of species at respective taxonomic levels (phylum, depicted in Figure 4A; and genus, depicted in Figure 4B) were evaluated and ranked. The taxonomic analysis revealed the existence of a single bacterial phylum (*p__Verrucomicrobiota*), one class, six bacterial orders, nine bacterial families, and eleven bacterial genera through the LEfSe multilevel species cladogram (Supplementary Figure 2) and linear discriminant analysis (Figure 4C). No statistically significant differences were observed in the relative abundances of these species at the phylum level, and fifteen distinct bacterial genera were identified at the genus level (Figure 4D). Eventually, eight genera (*g__norank_f__Eubacterium_coprostanoligenes_group*, *g__Romboutsia*, *g__Monoglobus*, *g__norank_f__Ruminococcaceae*, *g__dgA-11_gut_group*, *g__norank_f__Oscillospiraceae*, *g__unclassified_c__Clostridia*, and *g__norank_f__F082*) at the genus level were selected as the major effective bacteria between the SHC and MHC groups from the results of the linear discriminant analysis. Curated from the analysis’s results, these eight genera were selected as biomarkers of the intestinal microbiota.

**FIGURE 5:**
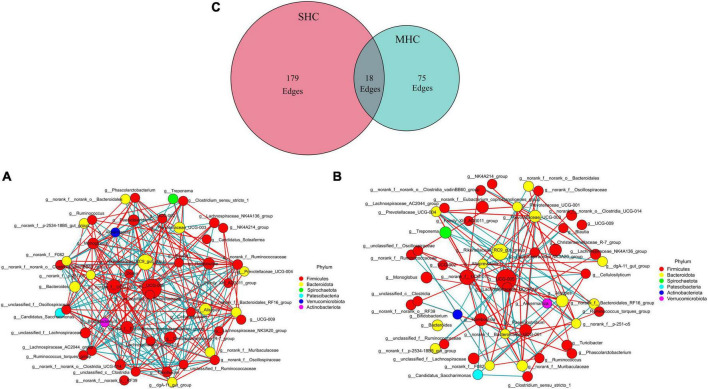
Interrelationship analysis within groups using co-occurrence network analysis. Displaying only significant (*P* < 0.05; *r* > 0.5) relationships. SHC group **(A)**: cows with slick and shining hair coat status, MHC group **(B)**: cows with rough and dull hair coat status, and common interactions are depicted in the Venn plot **(C)**. The nature of the relationship is depicted by red (positive) edges and blue (negative) edges. The size of the node corresponds with the mean abundance.

Using the same top 69 bacterial genera (relative abundance >0.1%) of the two groups, a co-occurrence network analysis (Figure 5) unveiled a total of 272 co-occurrence associations, with 102 positive relationships and 95 negative relationships within the SHC group (Figure 5A) and 48 positive relationships and 45 negative relationships in the MHC group (Figure 5B). Eighteen identical relationships were detected (Figure 5C). This outcome suggests varying composition, structure, and intra-group symbiotic relationships, with the disparity pointing to a higher degree of cooperation in the SHC group and increased competition within the MHC group. For example, *g__UCG-005*, the largest node in both groups, exhibited a positive correlation with five other genera and a negative correlation with eleven other genera in the SHC group. In contrast, *g__UCG-005* demonstrated a positive correlation with only one other genus and a negative correlation with eight other genera in the MHC group. In addition, the summed abundances of nodes in the two groups were different; the *g__norank_f__Eubacterium_coprostanoligenes_group*, *g__Christensenellaceae_R-7_group*, *g__Monoglobus*, *g__norank_f__norank_o__Clostridia_UCG-014*, *g__Ruminococcus*, and *g__Lachnospiraceae_NK3A20_group* in the SHC group showed higher value compared to the MHC group, while *g__Romboutsia*, *g__Prevotellaceae_UCG-003*, *g__Paeniclostridium*, *g__Treponema*, and *g__Prevotellaceae_UCG-004* in the SHC group presented lower value than in the MHC group (Supplementary Table 3).

**FIGURE 6:**
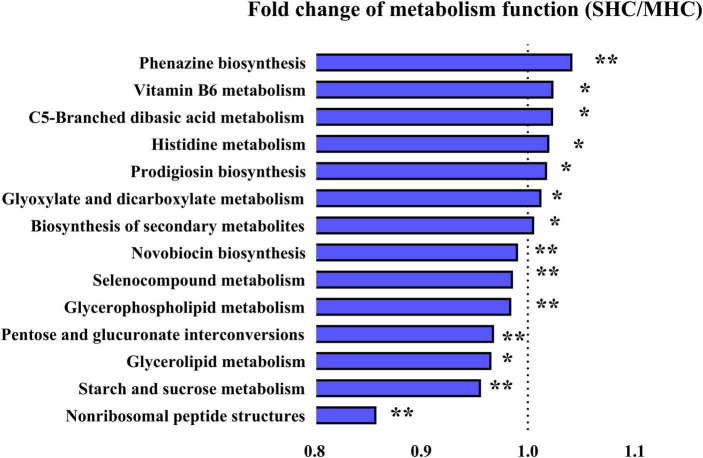
Illustration of detected differences in metabolic functions at the Level 3 of the KEGG pathway using PICRUSt. SHC: cows with slick and shining hair coat status; MHC: cows with rough and dull hair coat status. Differences were defined as significance with *P* < 0.05; *P < 0.05; **P < 0.01.

The functional analysis of the bovine intestinal microbiota (via KEGG pathways, level 3) revealed fourteen significant metabolic function pathways differing between the two groups (refer to Figure 6). These pathways consist of carbohydrate metabolism (starch and sucrose metabolism, glyoxylate and dicarboxylate metabolism, C5-branched dibasic acid metabolism, and pentose and glucuronate interconversions), amino acid metabolism (histidine metabolism and selenocompound metabolism), lipid metabolism (glycerophospholipid metabolism and glycerolipid metabolism), biosynthesis of other secondary metabolites (prodigiosin biosynthesis, novobiocin biosynthesis, and phenazine biosynthesis), metabolism of cofactors and vitamins (vitamin B6 metabolism), metabolism of terpenoids and polyketides (non-ribosomal peptide structures), and global and overview maps (biosynthesis of secondary metabolites). It was also discovered that functional enrichment in the immune system, endocrine system (including progesterone-mediated oocyte maturation and the estrogen signaling pathway), and replication and repair were more prevalent in the SHC group (refer to Supplementary Table 4). The observations suggest these pathways could indeed be beneficial to bovine pregnancy outcomes and overall health.

### 3.5 Intestinal microbe–host metabolite interactions associated with cattle hair coat status

In an effort to elucidate the interplay between gut microbiota and host metabolites, we executed a Procrustes analysis concerning the intestinal microbiome and plasma metabolome. An intriguing dissociation effect became evident in the MHC group (*P* = 0.066; Supplementary Figure 3), a pattern not replicated in the SHC group (*P* = 0.007; Supplementary Figure 3). To further explore the intricate interconnection among specific entities, we applied an additional network topological analysis, utilizing eight biomarker genera identified within the intestinal microbiota. This investigation revealed thirteen significantly different macromolecule metabolites and six biomarker small-molecule metabolites in the plasma metabolome. Subsequently, nodes were classified as either hubs or non-hubs, contingent upon their degree, closeness centrality, and betweenness centrality as gauged by thenetwork topological analysis.

**FIGURE 7:**
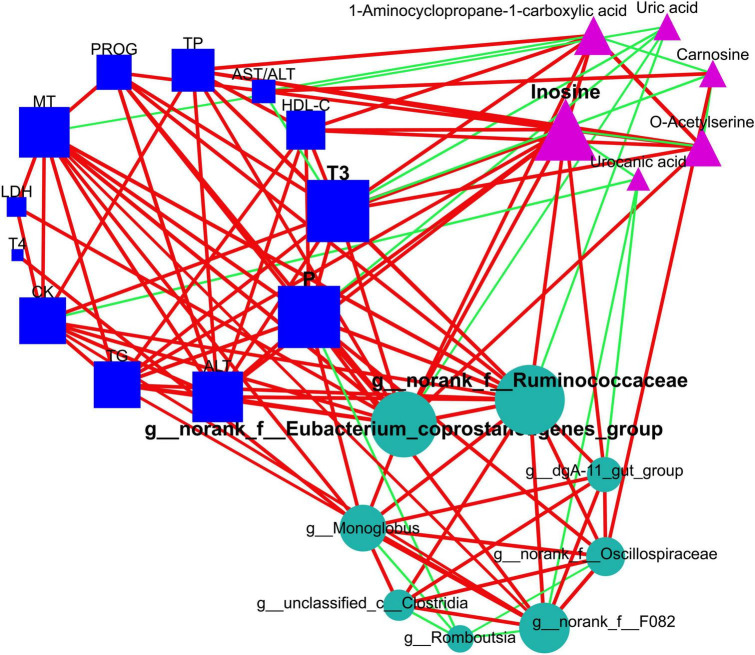
Elucidation of interactions among intestinal bacteria, plasma metabolome (small-molecule metabolites), and plasma macromolecule metabolites using network topological analysis. The plasma metabolome constitutes pathways for purine metabolism (inosine and uric acid), cysteine and methionine metabolism (1-aminocyclopropane-1-carboxylic acid and o-acetylserine), and histidine metabolism (urocanic acid and carnosine). The circles, triangles, and squares denote intestinal microbiome, plasma metabolome, and plasma macromolecular metabolites, respectively. The green edges suggest negative relationships, while red edges correspond to positive ones. Node sizes are proportional to the degree, closeness centrality, and betweenness centrality, and they are emphasized if identified as hubs within the networks. TG, triglycerides; HDL-C, high-density lipoprotein cholesterol; TP, total protein; AST, aspartate aminotransferase; ALT, alanine aminotransferase; CK, creatine kinase; LDH, lactate dehydrogenase; P, phosphorus; T3, triiodothyronine; T4, tetraiodothyronine; MT, melatonin; PROG, progesterone.

**FIGURE 8:**
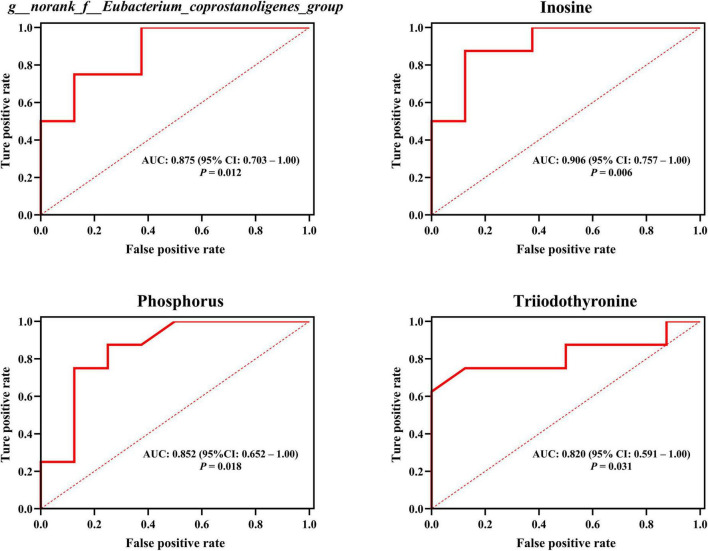
Employing plasma metabolites and intestinal bacteria to anticipate host hair coat status, as interpreted by the area under the receiver operating characteristic curve (AUROC). CI, confidence interval. Differences were defined as significance with *P* < 0.05.

Remarkably, our outcomes indicated that T3 and phosphorus (plasma macromolecule metabolites of plasma), inosine (plasma small-molecule metabolites), plus *g__norank_f__Eubacterium_coprostanoligenes_group* and *g__norank_f__Ruminococcaceae* (intestinal microbial genera) emerged as the central (Figure 7 and Supplementary Table 5), and these hubs were correlated (*r* > 0.5; *P* < 0.05). Venturing beyond this, we assessed the predictive potential of these hubs relative to host hair coat status by employing an AUROC curve analysis (Supplementary Table 6). Notably, we discerned that inosine, T3, phosphorus, and *g__norank_f__Eubacterium_coprostanoligenes_group* could effectively differentiate cows based on their hair coat status (AUROC value > 0.70, *P* < 0.05; Figure 8).

## 4 Discussion

The hair coat status of cattle serves as a readily observed indicator widely employed in animal production. Despite its longstanding application, the precise mechanism governing hair coat status in cattle remains largely unexplored. Our study supported our hypothesis, providing a potential mechanism whereby the intestinal microbiota relates to host metabolism, consequently impacting the hair coat status of cattle, using a late pregnancy Simmental cow model. These findings align with the reported intestinal microbiota-host interplay influencing hair status in humans and the existing intestinal biota-skin axis affecting skin conditions, such as alopecia areata (Mahmud et al., 2022).

Firstly, the SHC group cows exhibited a noticeably higher community diversity (α and β diversity) within their intestinal microbiota was detected. This observation supports the notion that increased diversity within an ecosystem can improve its stability, productivity, and overall functioning (Free et al., 2018). Furthermore, studies have revealed a correlation between a higher growth rate and enhanced diversity in the intestinal microbial community of human infants (Ford et al., 2019). Consequently, the observed greater diversity observed in the microbial community corresponds with the superior phenotypic hair coat condition of the host. On a related note, the host-specific properties of intestinal microbial communities properties when attempting to establish meaningful correlations between the host’s health and the occurrence or prevalence of particular microbial populations. This highlights the significance and of utilizing advanced, high-throughput approaches to examine the diversity and functionality of the gastrointestinal tract microbiota, a concept reinforced in a previous investigation (Zoetendal et al., 2008).

Secondly, an analysis of symbiotic relationships among intestinal microbiota revealed a higher level of cooperation in the SHC group in comparison to the MHC group. Within bacterial communities, competitive interactions are defined as two species utilizing shared resources, while cooperative interactions are defined as the metabolites produced by one species being consumed by another (potentially in a mutual manner), and cooperative loops result in direct and indirect benefits to all species involved and, consequently the host (Freilich et al., 2011). In fact, a lack of cooperative interactions causes microbiomes to be dysbiotic, and dominant organisms and low community diversity often indicate pathogenic human diseases (Welp and Bomberger, 2020; Chen Y. T. et al., 2021). Satiating this point, earlier studies have detected a higher degree of cooperation and less competition within the rumen microbiota of high-feed-efficiency dairy cows compared to their low-feed-efficiency counterparts (Xue et al., 2022). Apart from the inter-group interactions of intestinal microbiota, a Procrustes analysis revealed the presence of a disrupted symbiotic relationship between the host and the intestinal microbiota in the cows from the MHC group. This disruption may reflect competitive metabolism within the microbiomes for the metabolites intended to nourish the host, subsequently leading to a diminished symbiotic balance. Supporting this hypothesis are numerous examples where the disruption of the symbiotic balance results in an inferior phenotype or disease (Ramezani and Raj, 2014; Chen Y. T. et al., 2021). Functional analysis of the intestinal microbiota revealed fourteen significant variations in metabolic function pathways; for example, histidine metabolism was found to be enriched in the SHC group. These pathways indicate an intertwined function between the intestinal microbiota and the host hair coat status, as a higher level of histidine metabolism was notably found in the SHC group in the plasma metabolome. Moreover, we also discovered functions related to the endocrine system (namely, progesterone-mediated oocyte maturation and estrogen signaling pathway) were enriched in the SHC group via the PICRUSt analysis. These findings were in line with the elevated PROG concentrations observed in plasma within the SHC group, which could potentially have favorable impacts on the cows’ pregnancies. Additionally, hosts with different hair coat status had an intestinal bacteria response in some potentially beneficial bacteria. For example, *Akkermansia*, a mucus-degrading bacteria, is known to colonize mucus and has been implicated in anti-inflammatory activity, obesity, as well as regulating the immune response and metabolic functions of the intestinal and circulatory systems (Zhao et al., 2023). Our LEfSe analysis indicated that this genus and other genera from the phylum *Verrucomicrobia* were enriched in the SHC group, which in agreement with the findings reported in earlier studies. Notably, significant reductions in both *Akkermansia* abundance and diversity have been observed in human skin lesions in Olejniczak-Staruch et al. (2021).

Analysis of macromolecule metabolites in plasma precipitated by conventional testing methods, delineated specific indices linked to standard physiological changes and enriched metabolism in the SHC group in contrast to the MHC group. The enriched metabolic indices that exhibited higher readings encompassed lipids (TG and HDL-C), protein, liver function (AST and AST/ALT), cardiac function and energy metabolism (creatine kinase and lactate dehydrogenase), mineral element (phosphorus), nutritional hormones (T3, tetraiodothyronine, and melatonin), accompanied by a reproductive hormone (progesterone). On the aggregate, it can be surmised that the SHC group of cows may have demonstrated a superior performance relative to the MHC group in reproductive execution and offspring birth weight, congruent with findings from previous research (Foster et al., 2016; Poole et al., 2019). T3, as elucidated in earlier reviews, is a thyroid hormone pivotal to the management of energy metabolism, thermogenesis, fluid balance, hepatic nutrient metabolism, alongside the cardiovascular system (Ortiga-Carvalho et al., 2016). The thyroid hormone is also influential in spurring hepatic fatty acid synthesis and their esterification to TG, and bolstering fatty acids oxidation (Mullur et al., 2014). This correlation could account for the relatively escalated TG contents observed in the SHC group and may be indicative of an ameliorated fatty acid metabolism. Elevated phosphorus content in the SHC group suggests advanced energy utility and transit as phosphorus contributes to the formation of adenosine phosphate, and constitutes a component of nucleic acids involved in cellular proliferation and differentiation (Engle et al., 2016). These instances of empirical evidence lend credence to the role of T3 and phosphorus as potential biomarkers to prognosticate hair coat status. Both T3 and phosphorus participate in energy expenditure and metabolism, as well as in the hepatic nutrient metabolism (Engle et al., 2016; Ortiga-Carvalho et al., 2016), with T3 instrumental in maintaining a positive phosphorus equilibrium (MacDonald et al., 2021). A recent study involving broiler chickens has noted that an augmented prevalence of the genus *Eubacterium_coprostanoligenes_group* was linked to increased intestinal phosphorus absorption, elevated metabolic processes for bone formation, and mitigated phosphorus excretion (Wang et al., 2020). Dietary phosphorus supplementation in ducks amplified the occurrence of the cecal bacterial genus *Eubacterium_coprostanoligenes_group* (Dai et al., 2018). It is well established that the intestinal microbiota has the aptitude for hydrolyzing compounds secreted into the bile, involving itself in metabolism, and modulating the quantity of T3 in the intestine (DiStefano et al., 1993). These outcomes reaffirm our discovery of the positive interconnection among the three hubs T3, phosphorus, and *g__norank_f__Eubacterium_coprostanoligenes_group*.

Small-molecule metabolites, pivotal in numerous biological processes, can have their roles elucidated via metabolomics (Wishart, 2019). In the present study, we identified six such metabolites (uric acid, o-acetylserine, urocanic acid, 1-aminocyclopropane-1-carboxylic acid, inosine, and carnosine) in the plasma metabolome, coinciding three metabolic pathways (histidine metabolism, cysteine and methionine metabolism, and purine metabolism). The amino acids histidine, cysteine, and methionine possess a vital function in shaping the structure of skin and hair, specifically emphasizing sulfur-containing amino acids. Serving as forerunner molecules for keratin synthesis, cysteine and methionine are integral to hair structure. It’s worth noting that a significant amount of cysteine, approximately 10–17% by weight, is present in hair and animal genes associated with sulfur metabolism demonstrate high expression (Chai et al., 2021). Accordingly, the detection of enriched cysteine and methionine metabolism pathways in the SHC group was anticipated. These intriguing results not only reflect the hair coat status phenotype but also illustrate the utility of using metabolomics. The small-molecule metabolites linked to these critical metabolic pathways have been identified to be engaged in these biological processes. Previous research has underscored the importance of cysteine and methionine metabolism for maternal and neonatal health (Cochrane et al., 2022). Such findings also corroborate the hair coat status phenotype along with future pregnancy outcomes observed in late pregnancy Simmental cows, as noted in this and earlier studies (Poole et al., 2019).

In our study, we observed a significant enrichment of histidine metabolism in the SHC group when compared to the MHC group, both in the plasma metabolome and intestinal microbiota. This finding suggests a clear correlation between changes in the intestinal microbiota and host metabolism. Hair coat status reflects the skin condition; consistent with the findings of this study, other research has reported an association between alterations in histidine metabolism and skin conditions like psoriasis and acne in mice, which are caused by excessive keratinocyte differentiation and/or chronic skin inflammation (Zhu et al., 2021). Our analysis revealed a heightened abundance of the metabolites carnosine and urocanic acid in the metabolic pathways of histidine for the MHC group, coupled with lower histidine metabolism. Carnosine (beta-alanyl-L-histidine) is a compound synthesized from histidine and beta-alanine. The latter can be sourced from dietary intake or uracil degradation in the liver and is acknowledged for its antioxidant properties (Boldyrev et al., 2013). The amplified presence of carnosine in the MHC group likely stemmed from the increased oxidative stress these cows faced when skin issues arose due to their inferior hair coat status (Kamr et al., 2022). There’s also a possibility that the MHC group exhibited a lower capacity for blood carnosinase hydrolysis. It’s been demonstrated that blood rapidly hydrolyzes most carnosine into histidine and beta-alanine. These components are then absorbed by muscles, where carnosine is synthesized (Holeček, 2020). Urocanic acid, another metabolite of interest, is a product of histidine deamination in the liver (Hug et al., 1998). Past research has yet to confirm a correlation between high levels of urocanic acid and/or histidine and conditions such as protein malnutrition, immunosuppression, and elevated inflammation in humans and animals (Hug et al., 1998; Hart and Norval, 2021). These collective findings contribute significantly to the broader context of understanding the complex metabolic interplay in animals.

Intriguingly, our study discovered an enrichment of purine metabolism within the plasma metabolome. This highlighted discrepancies in nucleic acid metabolism between the two groups. Remarkably, dysregulation in purine metabolism and presence of inosine have emerged as potential standalone diagnostic biomarkers for major depressive disorder in pediatric and adolescent populations, characterized by a significantly lower abundance in the unhealthy group (Zhou et al., 2019). Our findings demonstrated higher purine metabolism and an increased abundance of inosine in the plasma. Mirroring our results, research demonstrated lower purine metabolism and a diminished abundance of inosine in the serum of cows experiencing subclinical and clinical mastitis compared to their healthy counterparts (Wang et al., 2022). An extensive look into the intestinal microbiota indicated that elevated diversity and mutual symbiotic relationships within the intestinal microbiota could elucidate why there was a considerable elevation in the level of inosine in the SHC group compared to the MHC group. This is because the production of inosine by bacteria, such as Saccharomyces cerevisiae, has been verified (Daignan-Fornier and Pinson, 2019). In a past study, a manipulated low-competition environment facilitated by the use of antibiotics and upregulated strains of Saccharomyces cerevisiae resulted in an enhancement of host purine metabolism (Chiaro et al., 2017). In a recent report, inosine has been linked with a contributing role in energy expenditure within apoptotic brown adipocytes (Niemann et al., 2022). Additionally, inosine has been found to create anti-inflammatory effects by acting to deter the production or release of pro-inflammatory cytokines, thus playing a crucial role in managing inflammatory diseases (da Rocha Lapa et al., 2012). These cumulative discoveries bolster our results, which pinpoint inosine as a vital biomarker for the hair coat status of cattle.

Our study identified the biomarkers of intestinal microbiota, which included included *g__norank_f__Eubacterium_coprostanoligenes_group* as a potential predictor for hair coat status. The effects of this genus may involve the reduction of cholesterol (Freier et al., 1994), lipid and bile acid metabolism (Xie et al., 2022), maintenance of colonic mucus barrier function, and decreased systemic inflammation (Bai et al., 2021; Yang et al., 2021). Interestingly, this beneficial genus has also been reported to be enriched in fecal samples from high-productivity sows, compared to their low-productivity counterparts during late pregnancy (Shao et al., 2019), as well as in the rumen of high-yield cows receiving the same diet (Tong et al., 2018). These findings provide supporting evidence for our result that this genus could serve as a predictor for hair coat status. Moreover, certain disease conditions further support our findings. In a mouse model with non-alcoholic steatohepatitis induced by a methionine choline-deficient diet, researchers observed a significant reduction in the abundance of *Eubacterium_coprostanoligenes_group* (Ye et al., 2018). Additionally, classical homocystinuria patients, characterized by increased plasma levels of total homocysteine and methionine, exhibited an overrepresentation of the genus *Eubacterium_coprostanoligenes_group* (Rizowy et al., 2020). Our study also revealed the involvement of cysteine and methionine metabolism in hair coat status. Notably, we reported that *Eubacterium_coprostanoligenes_group* exhibited positive correlations with metabolites in the purine metabolism pathway (inosine) and a negative correlation with uric acid. Consistently, prior studies have also reported positive correlations between the *Eubacterium_coprostanoligenes_group* and purine metabolism in the feces of mice (Xie et al., 2022).

The *Eubacterium_coprostanoligenes_group* is renowned for its unique ability to morph cholesterol into coprostanol (also referred to as fecal sterols), a compound that cannot be reabsorbed into the bloodstream, consequently reducing overall blood cholesterol levels (Freier et al., 1994). Thus, the MHC group, who exhibited a relatively lower abundance of *Eubacterium_coprostanoligenes_group*, may have experienced diminished cholesterol-to-coprostanol conversion within the intestine, leading to the amplification of reabsorbed cholesterol. This loop of cholesterol reabsorption has been witnessed to escalade in mice when their gut microbiota is compromised by antibiotics (Le Roy et al., 2019). Consequently, the MHC group, which projected a diminished intestinal microbial diversity, may have accentuated cholesterol reabsorption compared to their SHC counterparts. This would then explain the absence of any significant discrepancy in plasma cholesterol observed between the two groups.

Information pertaining to the genus *Eubacterium_coprostanoligenes_group* remains scant at present, underscoring a pronounced need for further exploration into the bacteria associated with varying hair coat statuses. Moreover, the intersection between the skin microbiome and skin ailments has been mapped out over several years in humans and experimental animal models (Harris-Tryon and Grice, 2022). The interplay between the skin’s microbiome and the gut-skin axis has also begun to be untangled in certain studies (Sinha et al., 2021). These yet untapped avenues hold potential to shed fresh light on the hair coat status of cattle.

## 5 Conclusion

In conclusion, intestinal *g__norank_f__Eubacterium_coprosta noligenes_group*, plasma inosine, triiodothyronine, and phosphorus can serve as distinguishable markers cows’ hair coat status. This study has unveiled the relationships between the characteristics of the intestinal microbiota, metabolites, and host’s hair coat status, providing compelling evidence and a fresh direction (intestine microbiota–host interplay) for future research endeavors striving to comprehend the hair coat status of cattle.

## Data availability statement

The datasets presented in this study can be found in online repositories. The names of the repository/repositories and accession number(s) can be found below: NCBI - PRJNA982038, MetaboLights database - MTBLS8726.

## Ethics statement

The animal study was approved by the Institutional Animal Care and Use Committee at Inner Mongolia Agricultural University (Approval Number: NND2023100). The study was conducted in accordance with the local legislation and institutional requirements.

## Author contributions

DW: Conceptualization, Data curation, Formal analysis, Investigation, Methodology, Resources, Software, Writing – original draft, Writing – review and editing. PZ: Data curation, Investigation, Methodology, Resources, Software, Writing – original draft. CW: Conceptualization, Methodology, Supervision, Validation, Visualization, Writing – original draft, Writing – review and editing. SH: Data curation, Investigation, Methodology, Writing – original draft. HC: Formal analysis, Investigation, Methodology, Resources, Software, Validation, Data curation, Writing – original draft. AC: Conceptualization, Formal analysis, Funding acquisition, Methodology, Project administration, Resources, Supervision, Validation, Visualization, Writing – review and editing.

## Footnotes

1
http://www.genome.jp/kegg/
