# Supplementary material for: Differences in the intestinal microbiota and association of host metabolism with hair coat status in cattle
Source: Front Microbiol. 2024 Apr 22;15:1296602. doi: 10.3389/fmicb.2024.1296602 (PMC11071169; doi:10.3389/fmicb.2024.1296602)
Supplement: Supplementary file 1 [file Table_1.docx]

Supplementary Material

# Supplementary Figures and Tables

## Supplementary Figures

**
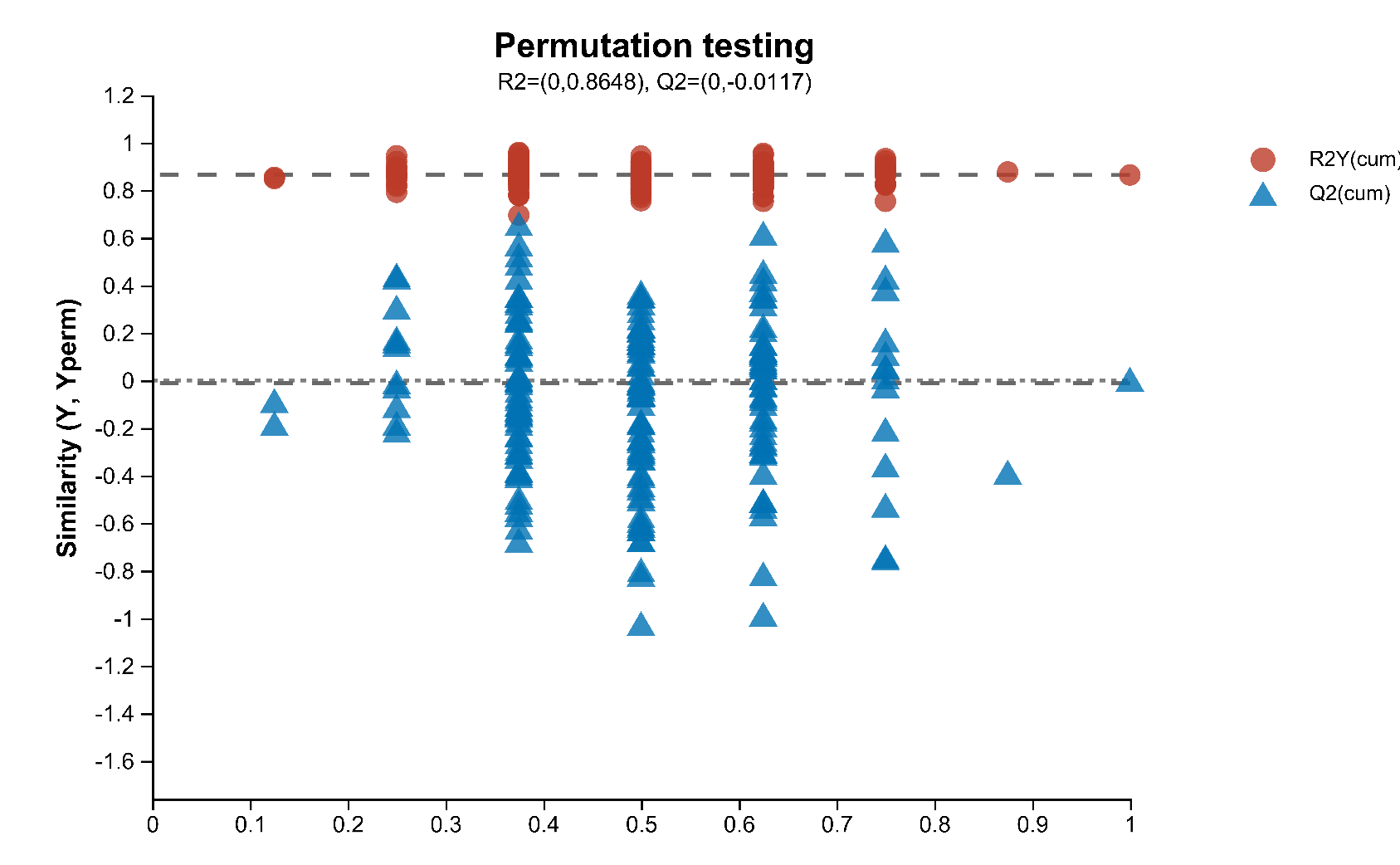
**

**
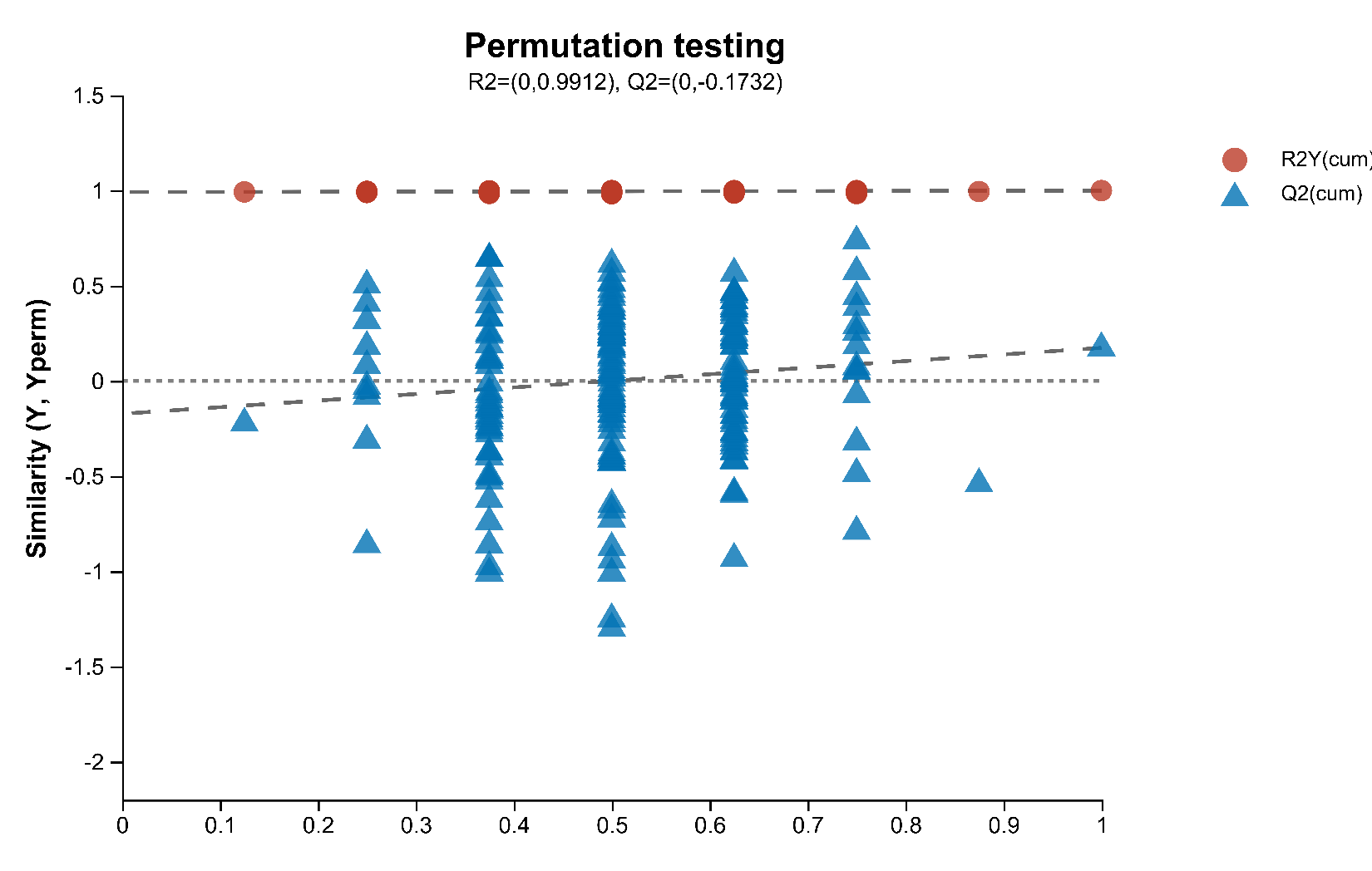
**

**Supplementary Figure 1.** The permutation test results for the Q2 intercepts positive and negative plasma samples of plasma metabolome. n = 8 for each group. Differences were defined as significance with *P* < 0.05.

**
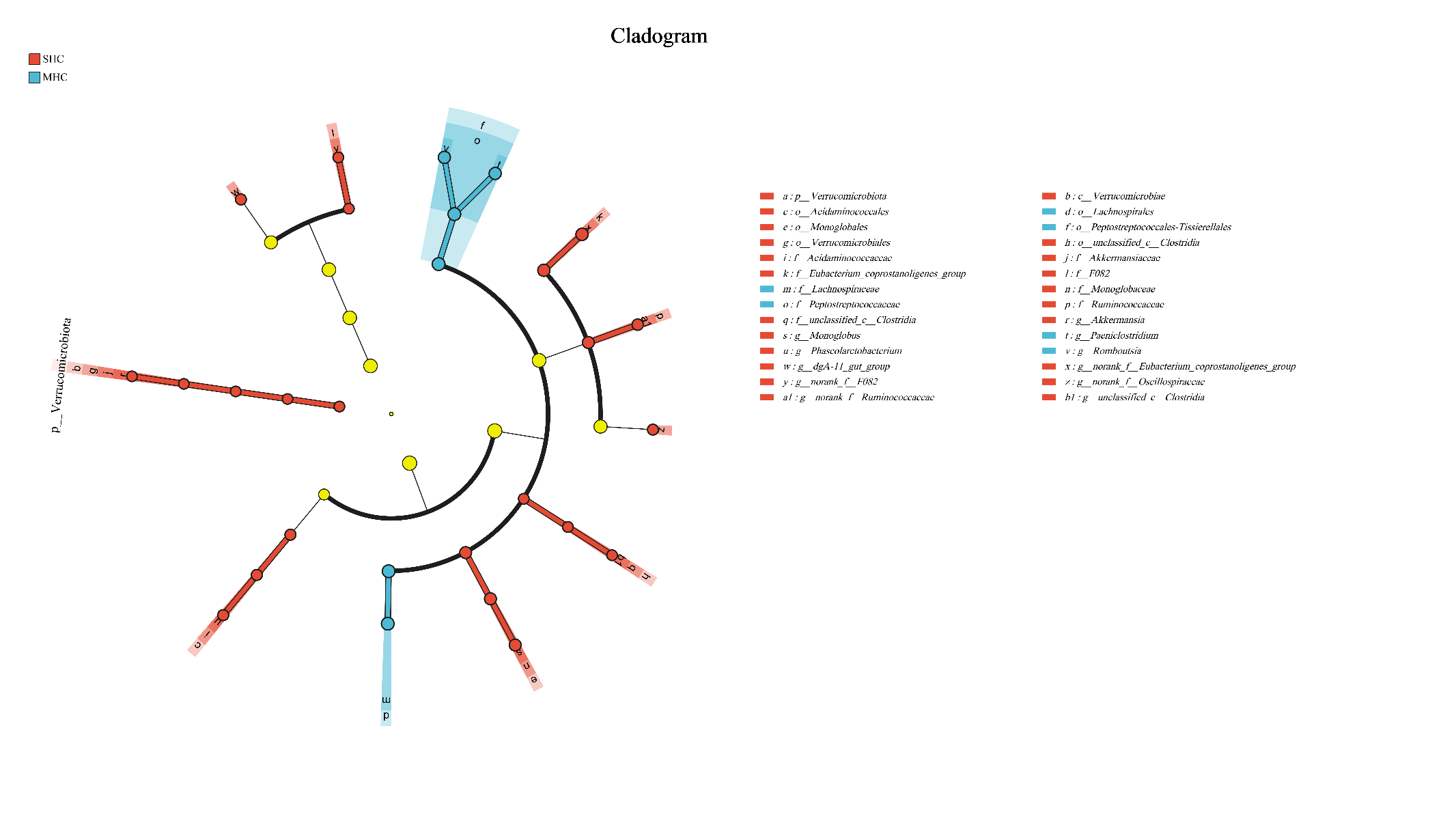
**

**Supplementary Figure 2.** Differential species of intestinal microbiota form phylum level to genus level using LEfSe multilevel species cladogram.

**
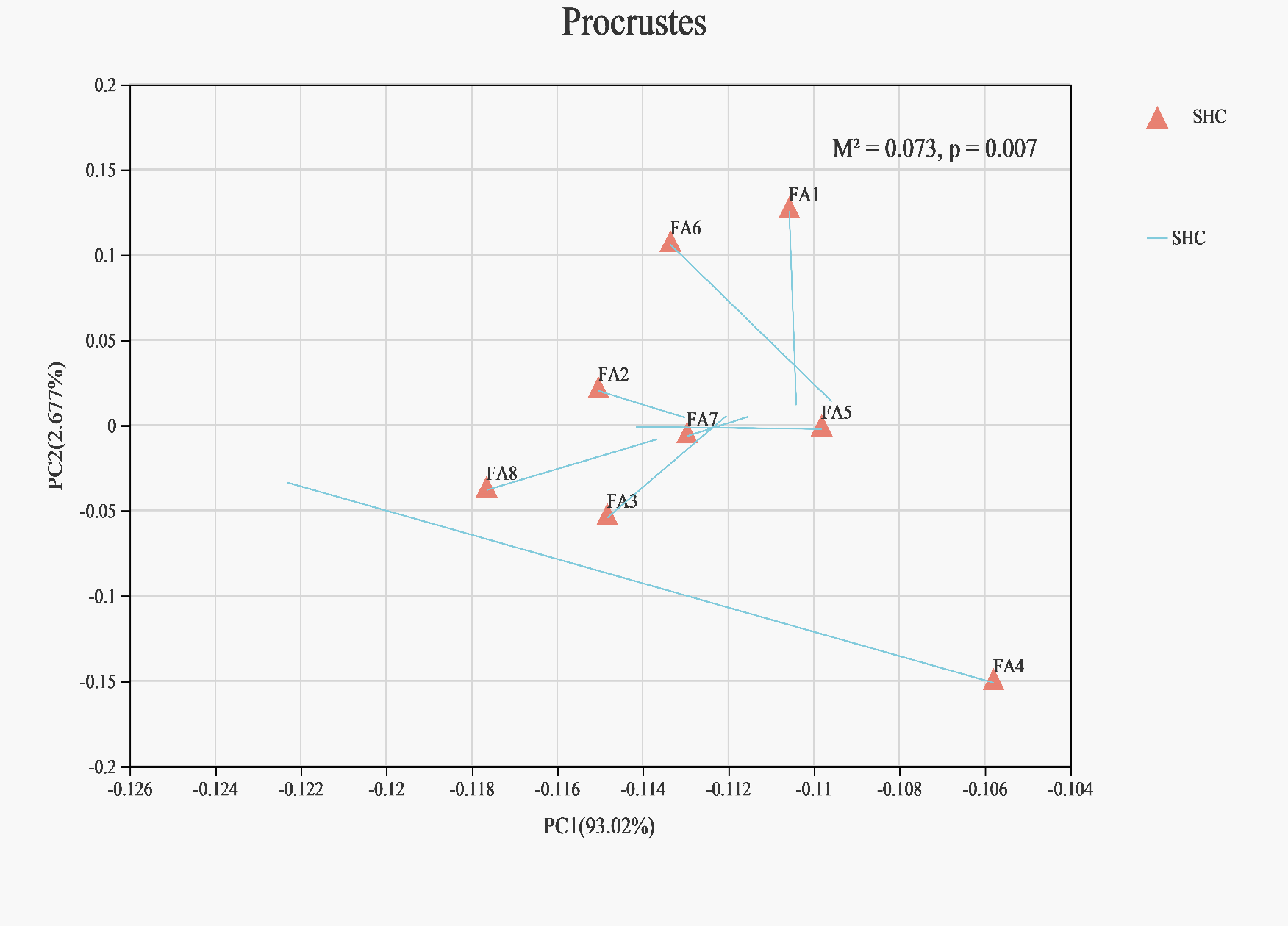
**

**
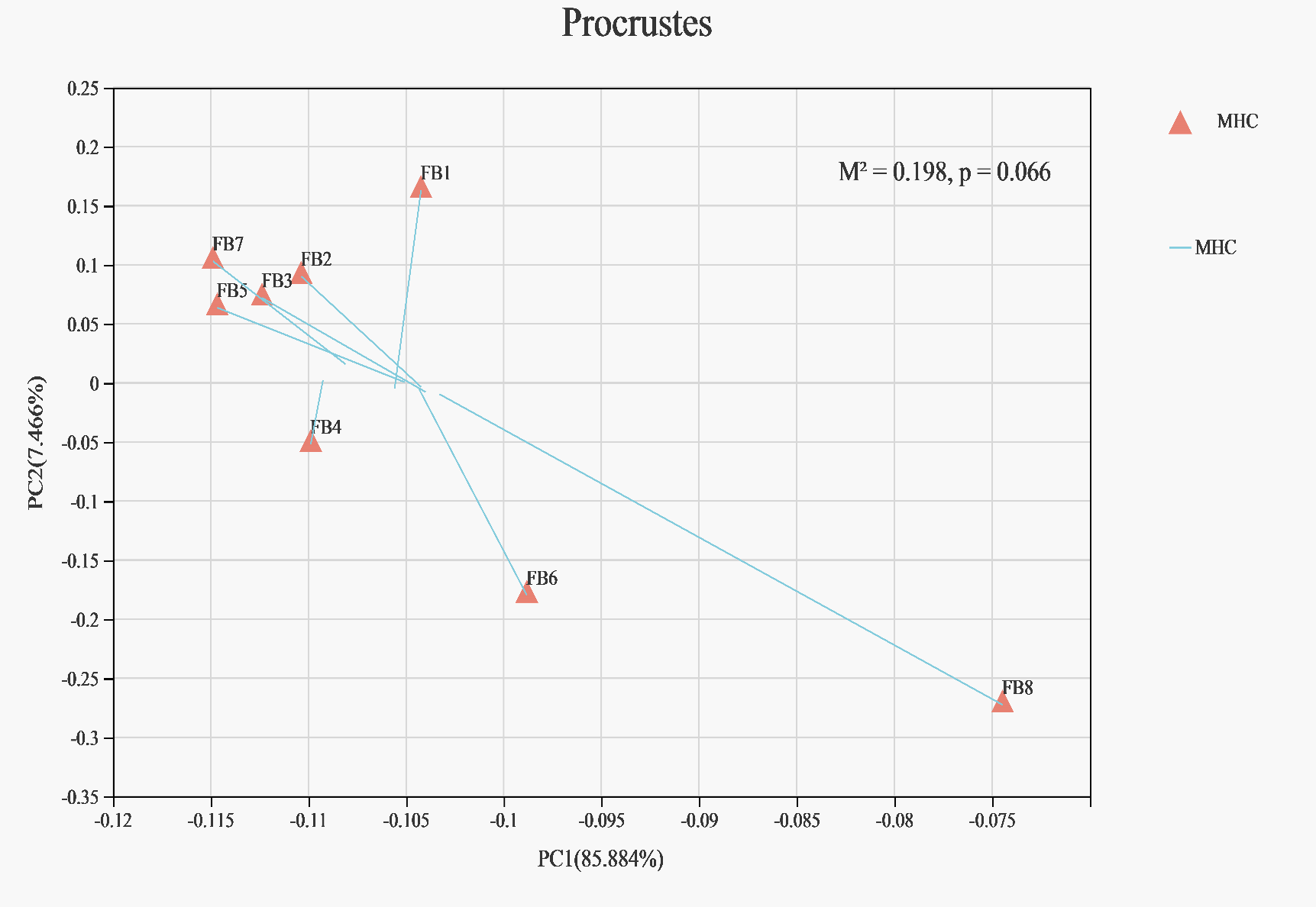
**

**Supplementary Figure 3.** Procrustes analysis for the intestine microbiome and plasma metabolome as cows of different hair coat status as slick and shining (SHC) or rough and dull (MHC). The line represents the intestine microbiome and the triangle represents the plasma metabolome.

## Supplementary Tables

**Supplementary Table 1.** Chemical compositions of the total mixed ration.

| Chemical analysis (DM, %) |  |
| --- | --- |
| Nutrients of % DM |  |
| Crude protein | 14.78 |
| Ether extract | 3.54 |
| Ash | 6.82 |
| Neutral detergent fiber | 48.72 |
| NFC^3^ | 26.14 |
| Acid detergent fiber | 12.36 |
| Starch | 16.23 |

^1^ DM = dry matter. The DM of the total mixed ration is 46.72%.

^2^ Composition of premix per kilogram: pantothenic acid 300 mg; niacin acid 300 mg; folic acid 30 mg; biotin 4 mg; I 12 mg; Se 12 mg; Co 12 mg; Fe 6,000 mg; Cu 1,000 mg; Zn 2,100 mg; Mn 2,100 mg; vitamin A 250,000 IU; vitamin D 150,000 IU; vitamin E 260,000 IU; vitamin K 90 mg; vitamin B1 32 mg; vitamin B2 120 mg; vitamin B6 50 mg; vitamin B12 0.5 mg.

^3^ Non-fiber carbohydrate (NFC) is calculated as 100 − (crude protein + ether extract + ash + neutral detergent fiber)

**Supplementary Table 2.** Information on sequencing depth of 16S rRNA genes.

| Sample ID | Total read bases (bp) | Clean reads | Mean length | Min length | Max length | Goods  -coverage |
| --- | --- | --- | --- | --- | --- | --- |
| SHC1 | 17859545 | 43286 | 412.594026 | 259 | 493 | 0.99455 |
| SHC2 | 21582195 | 52346 | 412.298838 | 270 | 441 | 0.994843 |
| SHC3 | 20036708 | 48632 | 412.006662 | 252 | 514 | 0.993599 |
| SHC4 | 18938618 | 45867 | 412.902915 | 245 | 444 | 0.993379 |
| SHC5 | 20482783 | 49517 | 413.651534 | 222 | 432 | 0.994879 |
| SHC6 | 22136441 | 53663 | 412.508451 | 278 | 498 | 0.993672 |
| SHC7 | 20358488 | 49456 | 411.648496 | 261 | 522 | 0.994111 |
| SHC8 | 18782554 | 45527 | 412.55857 | 293 | 447 | 0.994586 |
| MHC1 | 20361573 | 49387 | 412.286087 | 245 | 443 | 0.99455 |
| MHC2 | 17037871 | 41407 | 411.473205 | 216 | 432 | 0.994769 |
| MHC3 | 20612119 | 50036 | 411.945779 | 213 | 436 | 0.994184 |
| MHC4 | 22157263 | 53704 | 412.581242 | 277 | 432 | 0.99433 |
| MHC5 | 22774301 | 55278 | 411.995749 | 291 | 431 | 0.993526 |
| MHC6 | 18318957 | 44293 | 413.585826 | 261 | 431 | 0.994038 |
| MHC7 | 20647022 | 50170 | 411.5412 | 231 | 436 | 0.994294 |
| MHC8 | 20418265 | 49297 | 414.188794 | 265 | 432 | 0.993745 |
| Summation |  | 781866 |  |  |  |  |
| Average | 20156543.94 | 48866.625 | 412.4854609 | 254.9375 | 454 | 0.994190938 |

**Supplementary Table 3.** The order and sum abundance of the node of intestinal microbiota in the intra-group symbiotic relationship analysis using co-occurrence network analysis as cows of different hair coat status as slick and shining (SHC) or rough and dull (MHC).

| SHC | | | | MHC | | | |
| --- | --- | --- | --- | --- | --- | --- | --- |
| Order | Nodes | Feature type | Sum abundance | Order | Nodes | Feature type | Sum abundance |
| 1 | *g__UCG-005* | *Firmicutes* | 121.72 | 1 | *g__UCG-005* | *Firmicutes* | 118.11 |
| 2 | *g__Rikenellaceae_RC9_gut_group* | *Bacteroidota* | 75.44 | 2 | *g__Rikenellaceae_RC9_gut_group* | *Bacteroidota* | 72.78 |
| 3 | *g__norank_f__UCG-010* | *Firmicutes* | 61.13 | 3 | *g__norank_f__UCG-010* | *Firmicutes* | 60.16 |
| 4 | *g__norank_f__Eubacterium_coprostanoligenes_group* | *Firmicutes* | 40.88 | 4 | *g__Romboutsia* | *Firmicutes* | 35.18 |
| 5 | *g__Christensenellaceae_R-7_group* | *Firmicutes* | 34.18 | 5 | *g__Paeniclostridium* | *Firmicutes* | 30.49 |
| 6 | *g__Alistipes* | *Bacteroidota* | 29.19 | 6 | *g__Alistipes* | *Bacteroidota* | 29.25 |
| 7 | *g__Romboutsia* | *Firmicutes* | 27.51 | 7 | *g__Christensenellaceae_R-7_group* | *Firmicutes* | 28.91 |
| 8 | *g__Prevotellaceae_UCG-003* | *Bacteroidota* | 25.67 | 8 | *g__Prevotellaceae_UCG-003* | *Bacteroidota* | 28.23 |
| 9 | *g__Paeniclostridium* | *Firmicutes* | 23.84 | 9 | *g__norank_f__Eubacterium_coprostanoligenes_group* | *Firmicutes* | 28.22 |
| 10 | *g__Monoglobus* | *Firmicutes* | 23.44 | 10 | *g__Treponema* | *Spirochaetota* | 24.11 |
| 11 | *g__Bacteroides* | *Bacteroidota* | 19.42 | 11 | *g__Monoglobus* | *Firmicutes* | 20.11 |
| 12 | *g__unclassified_f__Lachnospiraceae* | *Firmicutes* | 16.39 | 12 | *g__unclassified_f__Lachnospiraceae* | *Firmicutes* | 18.02 |
| 13 | *g__norank_f__norank_o__Clostridia_UCG-014* | *Firmicutes* | 15.92 | 13 | *g__norank_f__Muribaculaceae* | *Bacteroidota* | 16.39 |
| 14 | *g__Turicibacter* | *Firmicutes* | 14.10 | 14 | *g__Turicibacter* | *Firmicutes* | 13.11 |
| 15 | *g__norank_f__Muribaculaceae* | *Bacteroidota* | 13.64 | 15 | *g__norank_f__Bacteroidales_RF16_group* | *Bacteroidota* | 12.50 |
| 16 | *g__norank_f__Bacteroidales_RF16_group* | *Bacteroidota* | 11.50 | 16 | *g__Lachnospiraceae_NK4A136_group* | *Firmicutes* | 12.09 |
| 17 | *g__norank_f__Ruminococcaceae* | *Firmicutes* | 11.47 | 17 | *g__norank_f__norank_o__Clostridia_UCG-014* | *Firmicutes* | 11.81 |
| 18 | *g__Lachnospiraceae_NK4A136_group* | *Firmicutes* | 11.35 | 18 | *g__norank_f__norank_o__Bacteroidales* | *Bacteroidota* | 10.18 |
| 19 | *g__Treponema* | *Spirochaetota* | 10.73 | 19 | *g__norank_f__norank_o__Clostridia_vadinBB60_group* | *Firmicutes* | 9.75 |
| 20 | *g__norank_f__norank_o__Bacteroidales* | *Bacteroidota* | 10.73 | 20 | *g__Prevotellaceae_UCG-004* | *Bacteroidota* | 9.39 |
| 21 | *g__norank_f__p-2534-18B5_gut_group* | *Bacteroidota* | 10.19 | 21 | *g__norank_f__Ruminococcaceae* | *Firmicutes* | 9.26 |
| 22 | *g__norank_f__norank_o__Clostridia_vadinBB60_group* | *Firmicutes* | 9.49 | 22 | *g__norank_f__norank_o__RF39* | *Firmicutes* | 9.10 |
| 23 | *g__Ruminococcus_torques_group* | *Firmicutes* | 7.52 | 23 | *g__norank_f__p-2534-18B5_gut_group* | *Bacteroidota* | 9.05 |
| 24 | *g__dgA-11_gut_group* | *Bacteroidota* | 6.98 | 24 | *g__Candidatus_Saccharimonas* | *Patescibacteria* | 7.33 |
| 25 | *g__norank_f__Oscillospiraceae* | *Firmicutes* | 6.44 | 25 | *g__Clostridium_sensu_stricto_1* | *Firmicutes* | 7.02 |
| 26 | *g__UCG-009* | *Firmicutes* | 6.24 | 26 | *g__Phascolarctobacterium* | *Firmicutes* | 6.76 |
| 27 | *g__Prevotellaceae_UCG-004* | *Bacteroidota* | 6.13 | 27 | *g__Ruminococcus_torques_group* | *Firmicutes* | 6.30 |
| 28 | *g__NK4A214_group* | *Firmicutes* | 6.02 | 28 | *g__dgA-11_gut_group* | *Bacteroidota* | 6.17 |
| 29 | *g__Clostridium_sensu_stricto_1* | *Firmicutes* | 5.78 | 29 | *g__norank_f__Oscillospiraceae* | *Firmicutes* | 5.53 |
| 30 | *g__Phascolarctobacterium* | *Firmicutes* | 5.66 | 30 | *g__NK4A214_group* | *Firmicutes* | 5.12 |
| 31 | *g__Ruminococcus* | *Firmicutes* | 5.23 | 31 | *g__Lachnospiraceae_AC2044_group* | *Firmicutes* | 5.07 |
| 32 | *g__norank_f__F082* | *Bacteroidota* | 4.83 | 32 | *g__unclassified_f__Oscillospiraceae* | *Firmicutes* | 4.97 |
| 33 | *g__unclassified_f__Oscillospiraceae* | *Firmicutes* | 4.67 | 33 | *g__unclassified_c__Clostridia* | *Firmicutes* | 4.26 |
| 34 | *g__Lachnospiraceae_AC2044_group* | *Firmicutes* | 4.38 | 34 | *g__norank_f__F082* | *Bacteroidota* | 3.77 |
| 35 | *g__unclassified_c__Clostridia* | *Firmicutes* | 4.21 | 35 | *g__Ruminococcus* | *Firmicutes* | 3.64 |
| 36 | *g__unclassified_f__Ruminococcaceae* | *Firmicutes* | 4.07 | 36 | *g__norank_f__p-251-o5* | *Bacteroidota* | 3.60 |
| 37 | *g__Lachnospiraceae_NK3A20_group* | *Firmicutes* | 4.05 | 37 | *g__Cellulosilyticum* | *Firmicutes* | 3.58 |
| 38 | *g__Family_XIII_AD3011_group* | *Firmicutes* | 3.43 | 38 | *g__Blautia* | *Firmicutes* | 3.31 |
| 39 | *g__Candidatus_Soleaferrea* | *Firmicutes* | 3.32 | 39 | *g__Prevotellaceae_UCG-001* | *Bacteroidota* | 3.19 |
| 40 | *g__UCG-002* | *Firmicutes* | 3.25 | 40 | *g__Family_XIII_AD3011_group* | *Firmicutes* | 3.05 |
| 41 | *g__Akkermansia* | *Verrucomicrobiota* | 3.20 | 41 | *g__UCG-002* | *Firmicutes* | 3.04 |
| 42 | *g__norank_f__p-251-o5* | *Bacteroidota* | 3.03 | 42 | *g__Bifidobacterium* | *Actinobacteriota* | 2.90 |
| 43 | *g__Bifidobacterium* | *Actinobacteriota* | 3.00 | 43 | *g__norank_f__Bacteroidales_UCG-001* | *Bacteroidota* | 2.88 |
| 44 | *g__Lachnospiraceae_UCG-010* | *Firmicutes* | 2.84 | 44 | *g__Akkermansia* | *Verrucomicrobiota* | 2.86 |
| 45 | *g__Cellulosilyticum* | *Firmicutes* | 2.41 | 45 | *g__Lachnospiraceae_NK3A20_group* | *Firmicutes* | 2.82 |
| 46 | *g__Blautia* | *Firmicutes* | 2.40 | 46 | *g__Alloprevotella* | *Bacteroidota* | 2.77 |
| 47 | *g__Dorea* | *Firmicutes* | 2.37 | 47 | *g__Lachnospiraceae_UCG-010* | *Firmicutes* | 2.37 |
| 48 | *g__unclassified_o__Oscillospirales* | *Firmicutes* | 2.32 |  |  |  |  |

**Supplementary Table 4.** Differences in non-metabolic functions at KEGG pathway level 3 were detected using PICRUSt.

| Pathway level 2 | Pathway level 3 | Fold Change (SHC/MHC) | SHC-Mean | MHC-Mean | SHC-Sd | MHC-Sd | *P*-value |
| --- | --- | --- | --- | --- | --- | --- | --- |
| Folding, sorting and degradation | Protein export | 1.008 | 0.632 | 0.627 | 0.00309 | 0.00393 | 0.013 |
| Folding, sorting and degradation | Protein processing in endoplasmic reticulum | 1.032 | 0.088 | 0.085 | 0.00106 | 0.00163 | 0.001 |
| Replication and repair | Non-homologous end-joining | 1.232 | 0.004 | 0.004 | 0.00065 | 0.00067 | 0.025 |
| Membrane transport | Bacterial secretion system | 1.019 | 0.565 | 0.554 | 0.00430 | 0.00731 | 0.004 |
| Membrane transport | Phosphotransferase system (PTS) | 0.840 | 0.149 | 0.178 | 0.01089 | 0.03155 | 0.030 |
| Immune system | Th17 cell differentiation | 1.014 | 0.047 | 0.047 | 0.00026 | 0.00071 | 0.026 |
| Immune system | IL-17 signaling pathway | 1.014 | 0.047 | 0.047 | 0.00026 | 0.00071 | 0.026 |
| Immune system | Antigen processing and presentation | 1.014 | 0.047 | 0.047 | 0.00026 | 0.00071 | 0.026 |
| Endocrine system | Progesterone-mediated oocyte maturation | 1.014 | 0.047 | 0.047 | 0.00026 | 0.00071 | 0.026 |
| Endocrine system | Estrogen signaling pathway | 1.014 | 0.047 | 0.047 | 0.00026 | 0.00071 | 0.026 |
| Cell growth and death | Apoptosis - fly | 1.091 | 0.034 | 0.031 | 0.00226 | 0.00222 | 0.024 |
| Cancer: specific types | Prostate cancer | 1.014 | 0.047 | 0.047 | 0.00026 | 0.00071 | 0.026 |
| Infectious disease: bacterial | Staphylococcus aureus infection | 0.712 | 0.011 | 0.016 | 0.00195 | 0.00538 | 0.041 |
| Neurodegenerative disease | Prion diseases | 0.758 | 0.004 | 0.005 | 0.00038 | 0.00081 | 0.002 |

Differences in compared data were defined as significance with *P* < 0.05; n = 8 for each group.

**Supplementary Table 5.** Properties of networks.

| Items | Degree | Closeness centrality | Betweenness centrality |
| --- | --- | --- | --- |
| Macro-molecule metabolites |  |  |  |
| T3 | 14 | 19.33 | 55.90 |
| P | 14 | 19.50 | 52.48 |
| MT | 11 | 17.67 | 22.01 |
| ALT | 11 | 17.83 | 7.06 |
| TG | 10 | 17.33 | 8.39 |
| CK | 10 | 17.00 | 19.30 |
| TP | 9 | 16.67 | 6.88 |
| HDL-C | 8 | 16.00 | 4.65 |
| PROG | 7 | 15.83 | 0.95 |
| AST/ALT | 4 | 12.75 | 0.00 |
| LDH | 3 | 12.67 | 0.00 |
| T4 | 1 | 10.42 | 0.00 |
| Small-molecule metabolites |  |  |  |
| Inosine | 14 | 19.33 | 48.01 |
| O-Acetylserine | 8 | 15.83 | 10.49 |
| 1-Aminocyclopropane-1-carboxylic acid | 8 | 15.83 | 10.49 |
| Uric acid | 5 | 14.67 | 0.66 |
| Carnosine | 5 | 14.17 | 11.67 |
| Urocanic acid | 4 | 13.83 | 1.23 |
| Bacteria |  |  |  |
| *g__norank_f__Ruminococcaceae* | 16 | 20.50 | 64.08 |
| *g__norank_f__Eubacterium_coprostanoligenes_group* | 15 | 20.00 | 67.03 |
| *g__norank_f__F082* | 11 | 17.50 | 27.28 |
| *g__Monoglobus* | 10 | 17.33 | 60.04 |
| *g__norank_f__Oscillospiraceae* | 8 | 16.33 | 22.74 |
| *g__dgA-11_gut_group* | 7 | 15.67 | 9.02 |
| *g__unclassified_c__Clostridia* | 6 | 14.67 | 1.07 |
| *g__Romboutsia* | 5 | 14.17 | 0.57 |

**Supplementary Table 6.** Plasma metabolites and intestinal bacteria to predict host hair coat status using the area under the receiver operating characteristic curves (AUC).

| Items | AUC | Confidence interval | | *P* value |
| --- | --- | --- | --- | --- |
| Macro-molecule metabolites |  |  |  |  |
| ALT | 0.906 | 0.757 | 1 | 0.006 |
| CK | 0.891 | 0.731 | 1 | 0.009 |
| P | 0.852 | 0.652 | 1 | 0.018 |
| MT | 0.828 | 0.606 | 1 | 0.027 |
| T3 | 0.820 | 0.591 | 1 | 0.031 |
| TP | 0.813 | 0.592 | 1 | 0.036 |
| TG | 0.805 | 0.579 | 1 | 0.041 |
| PROG | 0.797 | 0.575 | 1 | 0.046 |
| T4 | 0.773 | 0.527 | 1 | 0.066 |
| LDH | 0.766 | 0.528 | 1 | 0.074 |
| HDL-C | 0.742 | 0.491 | 0.993 | 0.104 |
| AST/ALT | 0.266 | 0.013 | 0.519 | 0.115 |
| GC | 0.156 | 0 | 0.39 | 0.021 |
| Small-molecule metabolites (plasma metabolome) |  |  |  |  |
| Inosine | 0.9063 | 0.7562 | 1 | 0.006 |
| Carnosine | 0.8438 | 0.6446 | 1 | 0.021 |
| 1-aminocyclopropane-1-carboxylic acid | 0.8125 | 0.5857 | 1 | 0.036 |
| Uric acid | 0.7656 | 0.5126 | 1 | 0.074 |
| O-acetylserine | 0.7656 | 0.5168 | 1 | 0.074 |
| Urocanic acid | 0.750 | 0.4964 | 1 | 0.093 |
| Bacteria (intestine microbiome) |  |  |  |  |
| *g__norank_f__Eubacterium_coprostanoligenes_group* | 0.875 | 0.703 | 1 | 0.012 |
| *g__norank_f__Ruminococcaceae* | 0.766 | 0.524 | 1 | 0.074 |
| *g__norank_f__F082* | 0.656 | 0.379 | 0.934 | 0.294 |
| *g__Monoglobus* | 0.656 | 0.340 | 0.972 | 0.294 |
| *g__norank_f__Oscillospiraceae* | 0.594 | 0.298 | 0.89 | 0.529 |
| *g__dgA-11_gut_group* | 0.641 | 0.359 | 0.922 | 0.345 |
| *g__unclassified_c__Clostridia* | 0.492 | 0.192 | 0.792 | 0.958 |
| *g__Romboutsia* | 0.406 | 0.11 | 0.702 | 0.529 |

Differences were defined as significance with *P* < 0.05.
